# Methylmercury Induced Neurotoxicity and the Influence of Selenium in the Brains of Adult Zebrafish (*Danio rerio*)

**DOI:** 10.3390/ijms18040725

**Published:** 2017-03-29

**Authors:** Josef Daniel Rasinger, Anne-Katrine Lundebye, Samuel James Penglase, Ståle Ellingsen, Heidi Amlund

**Affiliations:** National Institute of Nutrition and Seafood Research (NIFES), P.O. Box 2029 Nordnes, 5817 Bergen, Norway; anne-katrine.lundebye@nifes.no (A.-K.L.); sampenglase@hotmail.com (S.J.P.); stale.ellingsen@uib.no (S.E.); heidi.amlund@nifes.no (H.A.)

**Keywords:** contaminant, dietary, exposure, interaction, mechanisms, mercury, methylmercury, nutrient, proteomics, selenium

## Abstract

The neurotoxicity of methylmercury (MeHg) is well characterised, and the ameliorating effects of selenium have been described. However, little is known about the molecular mechanisms behind this contaminant-nutrient interaction. We investigated the influence of selenium (as selenomethionine, SeMet) and MeHg on mercury accumulation and protein expression in the brain of adult zebrafish (*Danio rerio*). Fish were fed diets containing elevated levels of MeHg and/or SeMet in a 2 × 2 full factorial design for eight weeks. Mercury concentrations were highest in the brain tissue of MeHg-exposed fish compared to the controls, whereas lower levels of mercury were found in the brain of zebrafish fed both MeHg and SeMet compared with the fish fed MeHg alone. The expression levels of proteins associated with gap junction signalling, oxidative phosphorylation, and mitochondrial dysfunction were significantly (*p* < 0.05) altered in the brain of zebrafish after exposure to MeHg and SeMet alone or in combination. Analysis of upstream regulators indicated that these changes were linked to the mammalian target of rapamycin (mTOR) pathways, which were activated by MeHg and inhibited by SeMet, possibly through a reactive oxygen species mediated differential activation of RICTOR, the rapamycin-insensitive binding partner of mTOR.

## 1. Introduction

Nutrients can modulate the toxic effects of contaminants [1]. For example, marine fatty acids have been shown to both ameliorate and exacerbate toxic insults from persistent organic pollutants [2,3]. Studies have also shown that co-occurring selenium and methylmercury (MeHg) reduce each other's toxic effects [4], and that dietary selenium may thus be able to explain the apparently contradictory results obtained from epidemiological studies in cohorts chronically exposed to low doses of MeHg [5].

Mercury is an environmental contaminant that causes toxic effects in humans and wildlife. Mercury is released to the marine environment from natural and anthropogenic sources, and is methylated to MeHg by microorganisms in sediments and the water column [6,7]. Methylmercury is readily bioavailable to fish and biomagnifies in the marine food chain, leading to higher levels of mercury in predatory fish [6,7]. In fish muscle, MeHg is predominantly present in the form of MeHg-l-cysteine (MeHg-l-Cys). Upon ingestion, gastrointestinal absorption of MeHg-l-Cys is high, and MeHg is rapidly distributed to all tissues, including the brain [8,9].

The neurotoxic effects of MeHg are well documented, and exposure to MeHg can lead to several adverse neurological effects [10]. In fish, the effects reported include perturbation of the brain proteome [11], reduced overall swimming activity, altered shoaling, and predator avoidance behaviour [12,13]. In humans, depending on the exposure level, paresthesia, dysarthria, progressive constriction of the visual field, hearing loss, sensory deficits, ataxia, tremor, deterioration of cognitive functions, and paralysis have been reported (reviewed in: [14]).

Selenium, an essential trace element utilised at the active site of a distinct set of proteins termed selenoproteins, is of considerable interest from both a toxicological and nutritional perspective [15]. Human health may be both beneficially and adversely affected by this metalloid and the European Food Safety Authority (EFSA) has set an Adequate Intake of 70 µg/day for adults [16]. Selenium is an abundant nutrient in fish, and a known antagonist of MeHg toxicity, however the underlying mechanisms are largely unknown. One hypothesis is that appropriate selenium levels uphold optimal antioxidant enzyme activities in cases of MeHg induced oxidative stress [17]. Another possible mechanism is the formation of MeHg-selenol complexes; elevated selenium levels may form MeHg-selenol complexes or lead to ligand exchange with MeHg-complexed thiol residues of proteins [18]. The MeHg-selenol complexes reduce the bioavailability of MeHg and possibly increase the excretion of MeHg from the brain [17,19].

Numerous experimental studies have investigated the molecular and cellular mechanisms of MeHg-induced neurotoxicity, but most work to date has focused on specific pathways and few report the systemic effects in the brain [20]. Proteomics analysis followed by functional and pathway analysis can generate new insights into cellular pathways and functions affected by different classes of toxicants, including metals [21]. For example, quantitative intact proteomics in Atlantic cod (*Gadus morhua*) brain highlighted mitochondrial dysfunction, oxidative stress, and altered calcium homeostasis as key target mechanisms of MeHg-induced neurotoxicity [11]. Similar pathways were also at the core of the biological functions affected in MeHg exposed mammalian models of toxicological research. For example, calcium signalling and transport pathways were among those most affected in the brain of MeHg exposed common marmoset monkeys [20]. However, in general proteomics studies analysing the effects of MeHg on the brain are scarce, and no proteomics profiling studies currently exist that examine the molecular effects of selenium on the brain proteome or the proposed selenium mediated modulation of MeHg neurotoxicity.

The present study used quantitative intact proteomic tools followed by bioinformatics pathway analyses to study the effects of dietary MeHg and selenium on the brain proteome in zebrafish (*Danio rerio*). The exposure experiments were performed using zebrafish as they are an important and well established research model for aquatic as well as human neuropharmacology and toxicology [22].

## 2. Results

### 2.1. General Observations and Accumulation of Mercury and Selenium

General observations and the influence of selenomethionine (SeMet) on the toxicokinetics of MeHg are reported elsewhere [23]. In brief, the overall performance of the zebrafish was good and the mortality was low and independent of dietary treatment. Mercury accumulated to the highest levels in the brain (60 ± 28 µg/g wet weight (ww), *n* = 3), followed by the liver (43 ± 13 µg/g ww, *n* = 3), and then muscle (6.4 ± 0.1 µg/g ww, *n* = 3) in zebrafish fed the MeHg diet. The relatively high accumulation of MeHg in the brain compared to the liver (1.4-fold) and muscle (9.4-fold) highlights the sensitivity of the brain to MeHg toxicity and the significance of MeHg as a neurotoxicant. Zebrafish fed the MeHg-SeMet diet had lower levels of mercury in the brain compared to those fed only MeHg (39 ± 9 vs. 60 ± 28 µg/g ww (*n* = 3), 35% lower). The mercury concentrations in the brain of zebrafish fed the control and SeMet diets were below the limit of quantification (LOQ). The selenium concentrations in all brain samples were below the LOQ.

### 2.2. Protein Expression Analysis and Protein Identification

**Figure 1:**
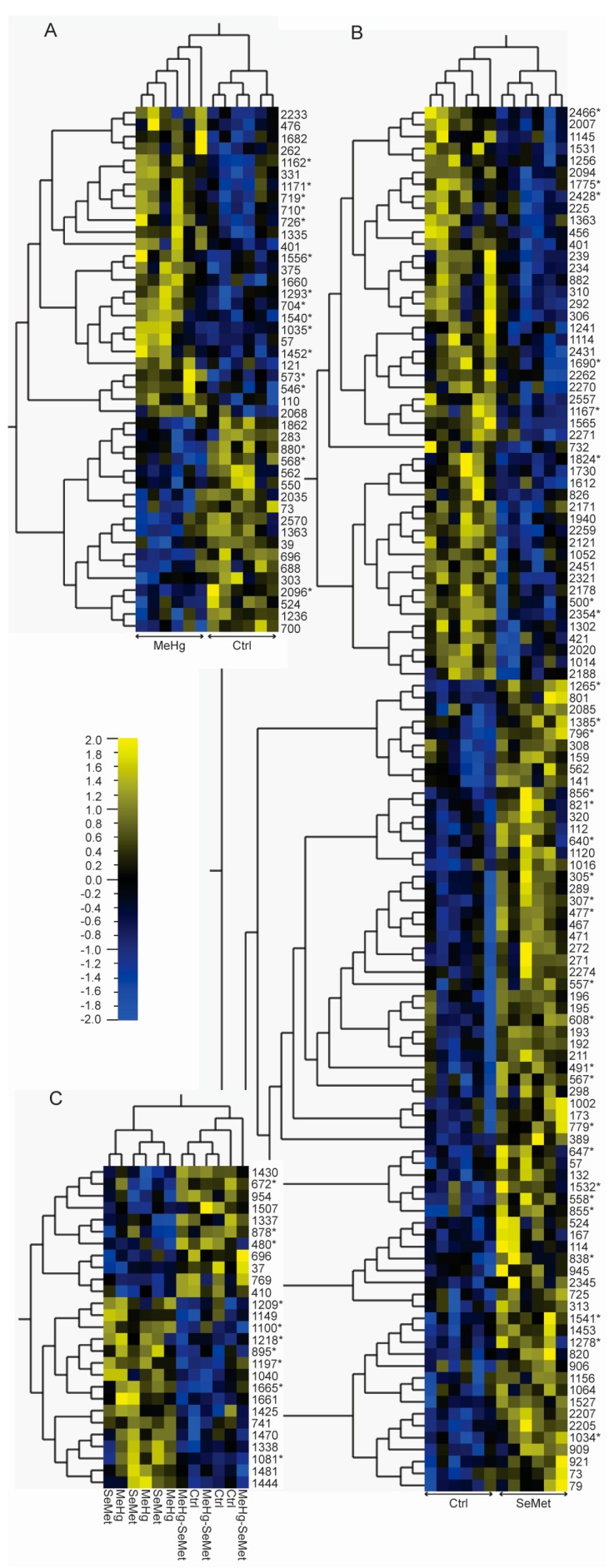
Heatmaps of significantly regulated (*p* < 0.05, 2-way ANOVA) proteins in the brain of zebrafish (*Danio rerio*). Zebrafish were fed control, methylmercury (10 µg Hg/g; MeHg), selenomethionine (5 µg Se/g; SeMet), or MeHg and SeMet (10 µg Hg/g + 5 µg Se/g) supplemented diets. After eight weeks of exposure, the brains (*n* = 12) were sampled and subjected to quantitative intact proteomics analysis. Differential analysis (2-way ANOVA) and hierarchical clustering (Pearson correlation) were performed using the Qlucore omics-explorer. (**A**,**B**) depict significantly differentially expressed proteins after exposure to MeHg and SeMet, respectively (*p* < 0.05, 2-way ANOVA); (**C**) Depicts the proteins displaying significant MeHg-SeMet interaction effects. The numbers in plots describe the unique difference in gel protein spot identifiers. Protein spots successfully identified by LC-MS/MS are denoted with an asterisk (*). See Table S1 for further details on individual proteins.

**Figure 2:**
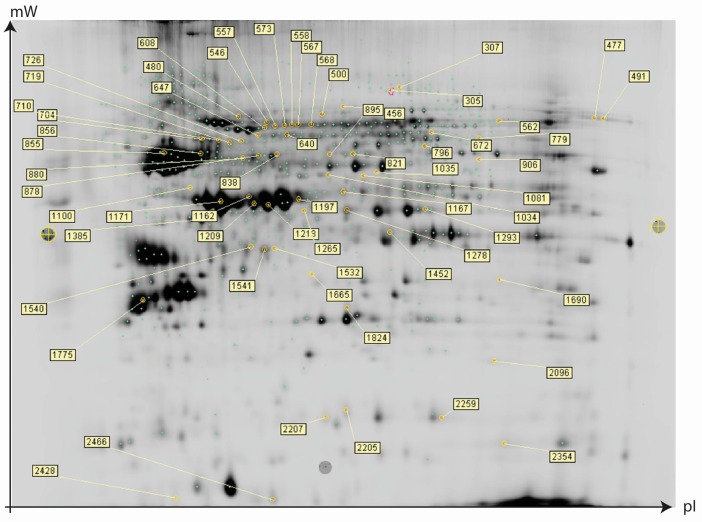
Preparative two-dimensional (2D) gel showing the picked and successfully identified significantly regulated (*p* < 0.05, 2-way ANOVA) proteins in brain of zebrafish (*Danio rerio*) after exposure to methylmercury (10 µg Hg/g; MeHg), selenomethionine (5 µg Se/g; SeMet) or MeHg and SeMet (10 µg Hg/g + 5 µg Se/g) supplemented diets. The numbers in the plot describe the unique difference in gel protein spot identifiers. The location of the spots relative to the x axis of the plot reflects the approximate isoelectric points of the protein spots. See Table S1 for further details on individual proteins.

Using Difference gel electrophoresis (DIGE) as previously described [24] followed by factorial ANOVA as implemented in the Qlucore Omics Explorer (version 2.3, Qlucore, Lund, Sweden), we detected 44 (26 up/18 down) and 116 (68 up/48 down) significant (*p* < 0.05) changes in the abundance of proteins in the brain of zebrafish fed the MeHg and SeMet diet, respectively (Figure 1A,B). In addition, we found 27 proteins, which displayed significant (*p* < 0.05) SeMet-MeHg interaction effects (Figure 1C). Focusing on the top ranked proteins reproduced successfully on preparative two-dimensional (2D) gels (Figure 2), a total of 64 protein spots were identified by liquid chromatography tandem mass spectrometry (LC-MS/MS). Relevant protein expression data including protein spot identifiers, statistical significance, fold changes, and protein identification features including accession numbers, protein names, isoelectric point, molecular weight, as well protein identification metrics are provided in Table S1.

### 2.3. Proteomics Bioinformatics and Pathway Analysis

**Figure 3:**
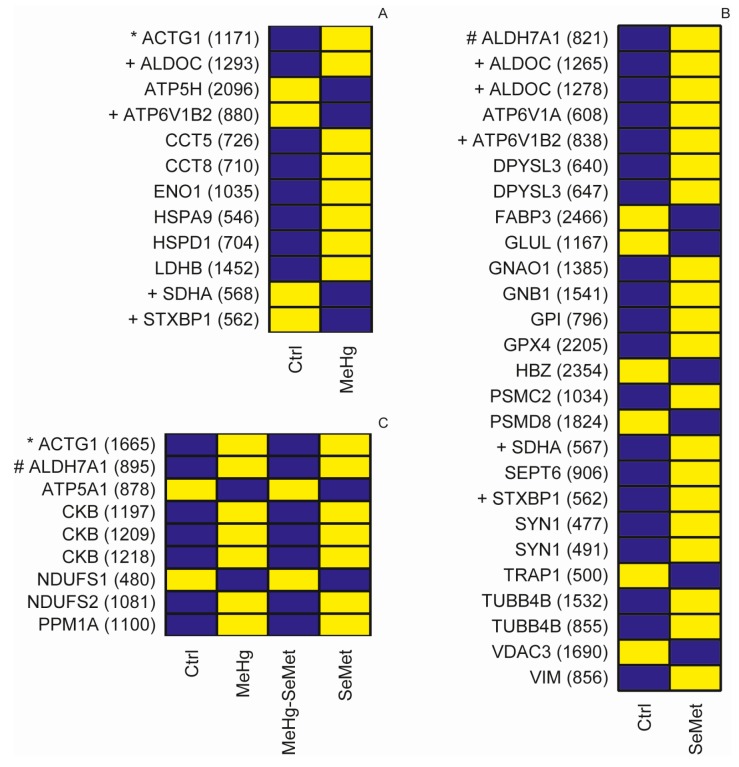
Heatmaps of significantly regulated (*p* < 0.05, 2-way ANOVA) proteins in the brain of zebrafish (*Danio rerio*). Significant protein responses elicited by: MeHg (**A**), SeMet (**B**) and MeHg-SeMet (**C**) interactions. The number in brackets following the Ingenuity Pathway Analysis (IPA) mapped identifier represent the gel master spot number provided in Figure 2 and Table S1, respectively. Individual proteins found to be regulated in response to (**A**,**B**) are labeled with a plus sign (+). Overlapping responses in (**A**,**C**) or (**B**,**C**) are marked with an asterisk (*) and a hash (#) sign, respectively. Yellow and blue boxes indicate increased and decreased expression levels relative to the treatment groups; the respective log2 fold changes are listed in Table S1.

**Figure 4:**
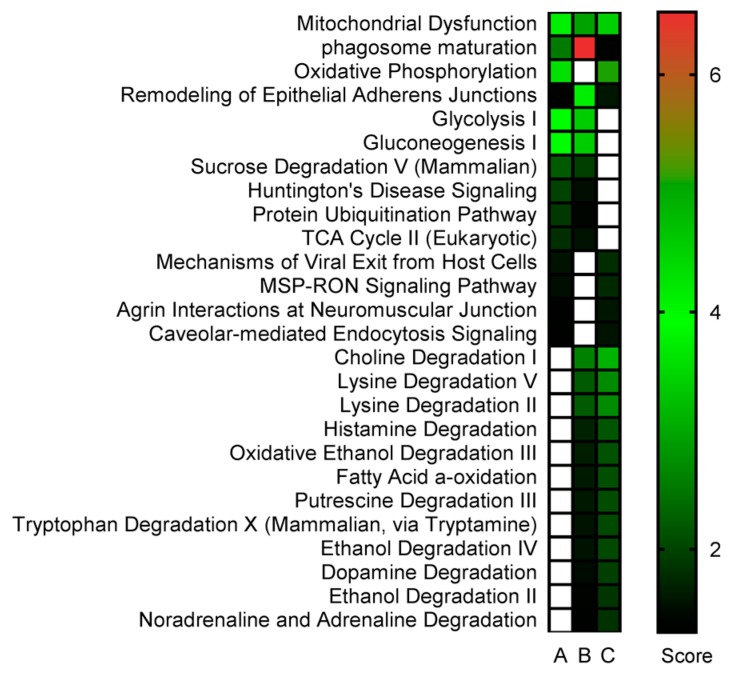
Canonical pathway analysis. Significantly regulated (*p* < 0.05, 2-way ANOVA) proteins in the brain of zebrafish (*Danio rerio*) after exposure to: MeHg (**A**), Se (**B**) and MeHg-SeMet (**C**). Interactions were subjected to IPA. Statistical significance of the overrepresentation of proteins in different “canonical pathways” is shown as a heatmap. Only selected pathways that were significantly (*p* < 0.05, Fisher’s exact test) enriched in at least one of the exposure conditions are shown alongside their significance values expressed as scores (−log10 *p*-value). Scores above the cut-off (1,3) are displayed by a color gradient. Scores below the cut-off value are displayed as white boxes. The full data-set including the proteins in each pathway is presented in Table S2B.

**Figure 5:**
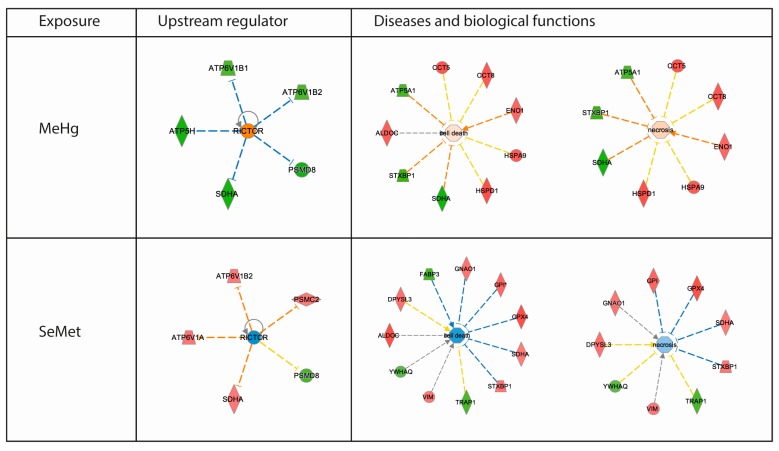
Ingenuity Pathway Analysis (IPA) of “upstream regulators” and “diseases and biological functions” of the lists of significantly regulated proteins (*p* < 0.05, 2-way ANOVA). RICTOR, the rapamycin-insensitive binding partner of the mammalian target of rapamycin (mTOR) was highlighted as a novel master upstream regulator that was predicted to be activated and inhibited in MeHg and SeMet exposed zebrafish brain, respectively. Diseases and biological functions analysis highlighted “cell death” and “necrosis” to be activated by MeHg and inhibited in SeMet exposed zebrafish brain. Activation (displayed in yellow) and inhibition (displayed in blue) of regulators and functions are based on IPA activation *z*-scores, which combine directional information encoded in the protein expression results with knowledge from the literature to make predictions about likely adverse outcome pathways. Up-regulated proteins are coloured red, down-regulated proteins are coloured green. The full data-sets of the upstream regulator analysis and the diseases and biological functions are presented in Table S2C,D.

The Ingenuity Pathway Analysis (IPA) platform was used with default settings to group proteins into larger functional categories (Table S2A–D). Successfully identified and IPA mapped proteins revealed both distinct and overlapping responses among the treatments (Figure 3). Similar to the differences observed in individual protein expression induced by the different diets, distinct and overlapping responses were also detected at the pathway level (Figure 4). Enrichment testing of the protein-lists against “diseases and biological functions” annotations in IPA revealed that MeHg toxicity and the modulation thereof by SeMet caused differential expression of proteins preferentially related to “cell death” and “survival” (Table 1). Causal network analysis in which the direction of changes induced in the zebrafish brain proteins was taken into account, highlighted that following exposure to MeHg the functions of “cell death” and “necrosis” appeared to be enhanced (*z*-score > 0) whereas after exposure to SeMet these two functions were predicted to be inhibited (*z*-score < 0; Table 1). To elucidate the root biological causes for the predicted effects on cellular and organismal biology, an upstream regulator analysis was performed. This analysis revealed that changes observed in the brain of zebrafish are linked to the mammalian Target of Rapamycin (mTOR) pathway, which was activated by MeHg and inhibited by SeMet through differential activation of RICTOR (rapamycin-insensitive companion of mTOR (mammalian target of rapamycin) (see Figure 5). Together, RICTOR and mTOR comprise a protein complex that integrates nutrient- and growth factor-derived signals to regulate cell growth.

## 3. Discussion

Selenium can influence the accumulation and toxicity of MeHg [25], but the underlying molecular mechanisms for this interaction remain poorly understood. To generate new insights into putative cellular pathways and functions that are affected, the present study compared and contrasted adverse outcome pathways of MeHg in the presence or absence of selenium, in the brains of dietary exposed zebrafish. The key findings of this study were: (1) MeHg exposure caused differential regulation of proteins in the zebrafish brain, which are associated with known molecular mechanisms underlying MeHg neurotoxicity; (2) SeMet exposure caused greater changes in brain protein expression than MeHg exposure; and (3) co-exposure of SeMet and MeHg reduced mercury accumulation in the brain and changed the brain proteome to a lesser degree than either MeHg or SeMet exposure alone.

The neurotoxic effects of MeHg have previously been investigated in vivo using proteomics approaches in fish, rodents, and monkeys [11,20,26,27]. This is the first study to use proteomics profiling tools on zebrafish as a model species to assess MeHg induced neurotoxicity. The MeHg induced brain proteome changes were similar in magnitude with those reported in other proteomics studies exploring MeHg exposure in the brain [11,20,26,27]. However, we found few similarities between this and other similar studies in relation to specific proteins affected by MeHg. More importantly, the changes detected in protein expression in the MeHg exposed zebrafish brain were, according to the functional analysis, indicative of several known adverse outcome pathways of MeHg exposure including mitochondrial dysfunction, oxidative stress, and the disruption of calcium homeostasis [19].

Interestingly, SeMet supplementation changed the protein abundances in the zebrafish brain to a greater extent than MeHg (116 vs. 44 proteins). Selenium exposure also had a greater effect on the magnitude of changes in the expression of proteins compared to MeHg. For example muscle cofilin 1 (CFL1) was upregulated 1.6-fold by SeMet exposure, while the greatest fold change induced by MeHg was −1.2-fold for ATP synthase. Several proteins that were predominately up-regulated by SeMet exposure in the current study were affected by MeHg exposure in other studies, where they were predominately downregulated. These include, for example, vimentin (VIM) and members of the tubulin (TUB), proteasome 26S subunit, non-ATPase (PSM), fructose-bisphosphate aldolase (ALDO), and glutathione peroxidase (GPX) families [26,27]. The three top ranked canonical pathways associated with the SeMet-induced proteome were also amongst those most affected by MeHg exposure. However, as exemplified by the biological functions of “cell death” and “survival”, the IPA activation-*z*-score indicated that SeMet exerted the opposite effect on protein expression to that induced by MeHg (see Table 1). This counteractive effect of SeMet on MeHg-induced protein regulation may help explain how selenium provides protection against MeHg-induced toxicity within cells.

Upstream regulator analysis further revealed that the changes observed in the brain proteome of zebrafish were linked to the differential activation of RICTOR. It is important to note that the RICTOR protein was itself not detected in the current study, possibly due to current limitations in gel-based proteomics which heavily favour the detection and quantification of highly abundant proteins. RICTOR defines a distinct mammalian mTOR pathway which regulates protein kinase C alpha (PKCα) and protein kinase B (Akt/PKB) signalling networks [28]. Within the mTOR pathway, the interplay between two protein complexes, mTORC1 and the RICTOR containing mTORC2, play an essential role in neural development [29]. While the mTOR pathway is complex and not completely understood, suppression and/or overactivation of either mTORC1 or mTORC2 results in dysregulation of neuronal morphology and function [30]. For example, the activation of mTOR signalling has been implicated in cadmium-induced neurotoxicity [31]. Like MeHg, cadmium is a heavy metal whose toxicity has been investigated as a possible etiological factor in neurodegenerative diseases.

Cadmium induced neurotoxicity is thought to be in part mediated through Reactive Oxygen Species (ROS)-induced activation of mTOR signalling [32]. Since the mTOR complexes are believed to be regulated by redox-based signalling mechanisms [28], it has been hypothesised that antioxidants may be exploited for the prevention of cadmium induced neurodegenerative diseases [32]. Rapamycin, a neuroprotective macrocyclic lactone, has been shown to prevent cadmium-induced neuronal cell death in vitro [31]. Additionally, cadmium-induced cell death, while not inhibited, was also significantly reduced in RICTOR-silenced PC12 cells [31], highlighting the importance of this regulator in the context of heavy metal toxicity in the brain. In addition to rapamycin, selenium was also reported to antagonise cadmium induced toxicity; most likely also through effects on the important balance between pro-oxidant toxic metals and antioxidant essential elements [33,34,35].

Beside the identification of proteins differentially expressed after exposure to either MeHg or SeMet, the factorial nature of the experiment allowed for the detection of proteins for which significant interactive effects occurred when MeHg and SeMet were co-exposed. In general, these proteins reflected a subset of the proteome that was regulated by SeMet or MeHg when administered separately, but whose dysregulation was prevented when both SeMet and MeHg were present. This counteraction may be due to SeMet and MeHg directly exerting a different effect on protein expression in each other’s presence. Alternatively, the direct binding of MeHg to SeMet in non-bioavailable complexes may have prevented either from having an effect on the redox balance and the proteome. Lower mercury levels in MeHg-SeMet compared to MeHg exposed zebrafish brains (35% less) may result in a concentration-dependent decrease in the effects of MeHg, and suggests that a direct interaction between MeHg and SeMet occurred. A lower accumulation of MeHg was also seen in the brain of zebra-seabream (*Diplodus cervinus*) when co-exposed with inorganic selenium [36]. However, in rats, selenium did not decrease MeHg accumulation in the brain, but still protected against MeHg-induced behavioural deficits [37]. This suggests that although SeMe decreased the mercury levels in zebrafish brains, it was not the only factor that allowed the MeHg-SeMet fish to maintain a brain proteome more similar to that found in unexposed fish. In addition, suppression of ROS modulated mTOR signalling pathways may contribute to the restoration of normal brain protein expression in MeHg SeMet co-exposed zebrafish. Further work is required to identify the mercury and selenium species present in brain tissue after exposure to MeHg and SeMet to elucidate the contribution of direct binding between mercury and selenium to the ameliorative effects of SeMet on MeHg-induced toxicity. Confirmatory experiments need to be run to assess the role of the mTOR pathways in the MeHg SeMet interactions.

## 4. Materials and Methods

### 4.1. Experimental Design and Sampling

Adult zebrafish (*Danio rerio*) were exposed to MeHg and/or SeMet as described elsewhere [23]. The experiment was approved by the Norwegian Animal Research Authority (now the Norwegian Food Safety Authority; approval number 2309; date of approval: 4 January 2010) and performed according to national and international ethical standards. In brief, zebrafish were housed in a multi-rack system for zebrafish (Aquatic Habitats Inc., Apopka, FL, USA) with continuously aerated and triple-filtered (mechanical, chemical (activated carbon), and biological) recirculating water (28.5 °C, pH 7.5, 500 µS/cm, and 10% daily water exchange). The photoperiod was 14 hours light and 10 hours dark. Zebrafish (males and females, average weight 0.32 ± 0.05 g, *n* = 27) were randomly distributed among 12 tanks (9 L), in a triplicate tank per treatment design and with 33–53 individuals in each tank. The zebrafish were fed three times a day, at a total ratio of 1.0% of their body weight daily. Zebrafish were fed the control, MeHg (10 µg Hg/g), SeMet (5 µg Se/g), or MeHg and SeMet (10 µg Hg/g + 5 µg Se/g, corresponding to a Se/Hg molar ratio of 1.26) supplemented diets for eight weeks.

The experimental diets were produced by adding aqueous solutions of MeHg and/or SeMet to a commercial pelleted zebrafish diet (Aqua Schwarz, Göttingen, Germany) as previously described [23]. Methylmercury was added to the diets as methylmercury-cysteine, while selenium was added as SeMet. The measured total mercury concentrations of the diets were 0.08 ± 0.01 µg/g (*n* = 3) for the control diet, 9.8 ± 0.5 µg/g (*n* = 3) for the MeHg diet, 0.16 ± 0.04 µg/g (*n* = 3) for the SeMet diet, and 9.3 ± 0.6 µg/g (*n* = 3) for the MeHg-SeMet diet. The measured selenium concentrations were 2.3 ± 0.1 µg/g (*n* = 3) for the control diet, 2.3 ± 0.1 µg/g (*n* = 3) for the MeHg diet, 4.3 ± 0.1 µg/g for the SeMet diet, and 4.3 ± 0.2 µg/g (*n* = 3) for the MeHg-SeMet diet. All diets were balanced with cysteine and/or methionine to equal the nominal levels in the MeHg-SeMet diet.

Zebrafish were sampled after eight weeks of exposure. The zebrafish were sampled randomly and euthanized immediately with an overdose of MS-222 (0.5 g/L; ethyl 3-aminobenzoate methane-sulphonate; Sigma-Aldrich, Copenhagen, Denmark). Zebrafish weights were recorded and the brains were excised. For proteomic analysis, individual samples were taken (*n* = 12) and immediately flash frozen in liquid nitrogen and then stored at −80 °C until further analyses. To guarantee sufficient tissue for measurements of mercury and selenium, the brains of *n* = 3 zebrafish from each tank were pooled. Samples were kept on ice during sampling and then stored at −20 °C until further analyses.

### 4.2. Mercury and Selenium Determination by Inductively Coupled Plasma Mass Spectrometry

Total mercury and selenium were determined by inductively coupled plasma mass spectrometry (ICP-MS) after microwave assisted decomposition as described elsewhere [38]. In brief, the samples were digested in 65% nitric acid (2 mL; Suprapur, Merck, Darmstadt, Germany) and 30% hydrogen peroxide (0.5 mL; Merck) using a microwave digestion system (Ethos 1600; Milestone, Sorisole, Italy). The solutions were diluted to 10 mL with deionised water (>17 MΩ/cm; Nanopure System; Barnstead, Dubuque, IA, USA). Total mercury and selenium concentrations were determined by ICP-MS (Agilent ICP-MS 7500c; Yokogawa analytical systems, Tokyo, Japan) equipped with an autosampler (ASX-500; CETAC Technologies, Omaha, NE, USA). Data were collected and processed using Agilent Chemstation ICP-MS software (version G1834B, Agilent Technologies, Palo Alto, CA, USA). Rhodium was used as an internal standard to correct for any drift of the instrument. The accuracy of the analytical method was assessed by the analysis of two certified reference materials; lobster hepatopancreas (TORT-2; National Research Council Canada, Ottawa, ON, Canada; for mercury; certified value 0.27 ± 0.06 µg Hg/g; obtained value 0.27 ± 0.00 µg Hg/g, *n* = 2; for selenium; certified value 5.63 ± 0.67 µg Se/g; obtained value 4.91 ± 0.18 µg Se/g, *n* = 2) and oyster tissue (SRM 1566b; National Institute of Standards and Technology, Gaithersburg, MD, USA; for mercury; certified value 0.0371 ± 0.0013 µg Hg/g; obtained value 0.032 ± 0.001 µg Hg/g, *n* = 4; for selenium; certified value 2.06 ± 0.15 µg Se/g; obtained value 2.05 ± 0.06 µg Se/g, *n* = 4). The LOQ of the method is 0.005 and 0.01 µg/g for mercury and selenium, respectively.

### 4.3. Proteomics Sample Preparation and Cydye Labeling

Unless otherwise stated, all chemicals and equipment used for DIGE were purchased from General Electrics (GE) Healthcare Life Sciences (Oslo, Norway). All DIGE related work was performed as previously described [24]. In brief, proteins were extracted using the Sample Grinding Kit with a standard 2× DIGE Lysis Buffer (7 M Urea, 2 M Thiourea, 4% *w*/*v* 3-[(3-Cholamidopropyl)dimethylammonio]-1-propanesulfonate hydrate (CHAPS), 2% *w*/*v* DL-Dithiothreitol (DTT), and 2% *v*/*v* PharmalyteTM pH 3–11). Lipids and nucleic acids were removed from the samples using the 2D Clean-up Kit. Protein pellets were re-suspended in DIGE Labeling Buffer (7 M Urea, 2 M Thiourea, 4% *w*/*v* CHAPS, 30 mM Tris). Following protein quantitation (2D Quant Kit), all samples were labelled (*n* = 12, 50 µg each) with 200 pmol of fluorescent cyanine dyes using either the minimal CyDyeTM DIGE Fluors Cy3 or Cy5 in a symmetrical dye-swap design. In addition, a master mix (internal standard, IS) of all samples mixed in a 1:1 ratio was prepared. A portion of the IS (300 µg) was labeled with 1.2 µmol Cy2 CyDyeTM for DIGE; the remainder (500 µg) was labelled with Lightning Red (Serva, Heidelberg, Germany).

### 4.4. Difference in Gel Electrophoresis

Randomly selected Cy3 and Cy5 labelled samples (three per exposure group) were combined with 50 µg of Cy2TM labeled IS and loaded onto immobilized pH gradient IPG strips (24 cm, pH 3–11 NL) using overnight rehydration loading. Isoelectric focusing (IEF) was performed on an Offgel Fractionator (Agilent Technologies) following the vendor’s instructions for conventional in-gel IEF. Upon completion of the IEF, IPG strips were equilibrated for 15 min in equilibration buffer (6 M Urea, 30% *v*/*v* glycerol, 2% SDS *v*/*v*, 50 mM Tris, and 0.02% *w*/*v* Bromophenol Blue) to which 1% *w*/*v* DTT was added. After 15 min of equilibration, this step was repeated using the same buffer as before, without DTT and 2.5% *w*/*v* iodoacetamide added. Polyacrylamide gel electrophoresis was performed using the Ettan DALTsix large format vertical system using precast DIGE gels. The gels were run at 0.5 W/gel for the first hour and at 15 W/gel until the Bromophenol Blue dye front had migrated off the lower end of the gels.

### 4.5. Image Acquisition and Analysis

Following the electrophoretic separation of the CyDyeTM-labelled proteins, the gels were imaged immediately using a Typhoon 9400 (GE Healthcare, Little Chalfont, UK). Acquired images were subjected to data analyses as previously described [24]. In short, spot maps were cropped and loaded into the Batch module of the DeCyderTM 2D (version 7.2, GE Healthcare) where a Differential In-Gel Analysis (DIA) was performed. The parameters for the spot detection algorithm were set manually to compensate for non-protein artefacts on the image (e.g., dust particles and scratches on the glass plates). Following the vendor’s instructions, the estimated number of spots was set to 10,000 and the spot volume cut-off to 30,000. Following DIA, normalised spot volumes were exported to the statistical programming language R (version 3.2.3, https://www.R-project.org/). In R, the bioconductor package Limma (version 2.4, https://www.bioconductor.org) was used to generate Bland-Altman (MA) plots of each spot map, which were created to assess the quality of each DIA-matched gel. The gel with the least observable bias, and an average number of detected spots, was chosen as the master gel for the Biological Variation Analysis (BVA). The master gel was also exported as a template gel to facilitate the matching of the analytical gels with the preparative gels. Upon completion of the BVA process, match qualities were assessed by visual inspection of each auto-level 1 match (i.e., the spots assigned the highest match quality) until a satisfactory number of matches were obtained that displayed high match-qualities across all areas of the gels.

Matched protein spots present on all spot maps were exported to Qlucore Omics Explorer (version 2.3, Qlucore) for differential protein expression analysis using factorial ANOVA. The number of differentially expressed proteins for each main treatment factor were calculated by establishing a set of orthogonal contrasts assessing (i) the main effect of MeHg; (ii) the main effect of SeMet; and (iii) the MeHg-SeMet interaction effects. The latter are the proteins which are differentially regulated due to the presence of both MeHg and SeMet, but whose expression levels may not necessarily be statistically significantly altered by one compound alone. A *p*-value cut-off of 0.05 was chosen as the significance threshold. Protein spots were considered for LC-MS/MS identification if in addition to reaching statistical significance, the spots were reproduced with confidence on preparative 2D gels.

### 4.6. Mass Spectrometry

Protein spots of interest were excised using a semi-automatic Screen Picker (Serva). Excised gel plugs were digested with sequencing grade trypsin (Promega, Southampton, UK) using an Investigator ProGest (Genomic Solutions, Ann Arbor, MI, USA) robotic digestion system [39,40]. Tryptic peptides were separated by reverse phase nano-flow liquid chromatography (Ultimate 3000, C18 PepMAp 100, 3 µm, 100A, 25 cm, ThermoFisher Scientific, Loughborough, UK) and subjected to online tandem mass spectrometry (MS/MS) analysis (LTQ Orbitrap XL, ThermoFisher Scientific, Loughborough, UK)) via a nano-spray source (Picoview, New Objective, Woburn, MA, USA). Spectra were collected from the mass analyser using full ion scan mode over the *m*/*z* range 450–1600. Six dependent MS/MS scans were performed on each ion using dynamic exclusion.

### 4.7. Bioinformatics

Standard bottom-up proteomics bioinformatics analysis was conducted using SearchGUI version 2.2.2 [41]. In short, Mascot Generic Format (MGF) Peak lists obtained from MS/MS spectra were identified using open mass spectrometry search algorithm (OMSSA) version 2.1.9, X!Tandem Sledgehammer (2013.09.01.1), Andromeda version 1.5.3.4, MS Amanda version 1.0.0.5242, MS-GF+ version Beta (v10282), Comet version 2015.02 rev. 3, MyriMatch version 2.2.140, and Tide.

Protein identification was conducted against a concatenated target/decoy version of the *Danio rerio* complement of the UniProtKB (version of 18.1.2016, 56379 (target) sequences). The decoy sequences were created by reversing the target sequences in SearchGUI. The identification settings were as follows: Trypsin with a maximum of 2 missed cleavages; 10.0 ppm as MS1 and 0.8 Da as MS2 tolerances; fixed modifications: Carbamidomethylation of C (+57.021464 Da), variable modifications: Oxidation of M (+15.994915 Da), Acetylation of protein N-term (+42.010565 Da), Pyrolidone from E (−18.010565 Da), Pyrolidone from Q (−17.026549 Da) and Pyrolidone from carbamidomethylated C (−17.026549 Da), fixed modifications during refinement procedure: Carbamidomethylation of C (+57.021464 Da).

Peptides and proteins were inferred from the spectrum identification results using PeptideShaker version 1.2.2 [42]. Peptide Spectrum Matches (PSMs), peptides, and proteins were validated at a 1.0% False Discovery Rate (FDR) estimated using the decoy hit distribution. All validation thresholds are listed in the Certificate of Analysis available in the supplementary information. Post-translational modification localizations were scored using the D-score and the phosphoRS score as implemented in the compomics-utilities package.

Biological network analysis was used to interpret the proteomics data obtained. Uniprot-Swissprot Accession numbers of identified differentially expressed proteins were imported into the Ingenuity Pathway Analysis software suite (IPA, Quiagen, Redwood City, CA, USA). Successfully mapped proteins were subjected to an IPA Core Analysis using default settings followed by targeted upstream regulator analysis.

## Figures and Tables

**Table 1:** Ingenuity Pathway Analysis (IPA) of significantly regulated (*p* < 0.05, 2-way ANOVA) proteins in the brain of zebrafish (*Danio rerio*). Statistical significance of the overrepresentation of genes in different “Diseases and biofunctions” is shown as “Score” (−log10 *p*-value, Fisher’s exact test). Only selected pathways for which in addition to statistical significance (Score > 1.3) activation *z*-scores were obtained in at least one of the exposure conditions are shown. Activation *z*-scores combine directional information encoded in the protein expression results with knowledge from the literature to make predictions about the activation or inhibition of likely adverse outcome pathways. The blue and yellow cells indicate whether the displayed pathways were predicted to be inhibited or activated, respectively. The full data-set including the proteins in each pathway is presented in Table S2C.

Diseases and Biofunctions	Score		Activation	
	MeHg	SeMet	MeHg	SeMet
Cell death and survival				
Cell death	3.0	1.8		
Cell death of neuroblastoma cell lines	6.6			
Cell death of tumor cell lines	2.8			
Necrosis	3.0	1.7		
Cell death and survival, neurological disease				
Neuronal cell death		2.4		
Cellular assembly and organization				
Formation of cytoskeleton		2.6		
Cellular assembly and organization, cellular function and maintenance				
Microtubule dynamics		1.9		
Organization of cytoskeleton		2.2		
Cellular assembly and organization, tissue development				
Formation of filaments		3.5		
Cellular compromise				
Degeneration of cells		3.9		
Cellular growth and proliferation				
Proliferation of cells		3.0		
Infectious diseases				
Viral infection		2.4		
Molecular transport				
Transport of molecule		2.4		
Neurological disease				
Degeneration of nervous system		3.1		
Neurodegeneration		3.0		
Organismal survival				
Organismal death		1.9		

## Supplementary Materials

Supplementary materials can be found at www.mdpi.com/1422-0067/18/4/725/s1.

## Abbreviations

IPA	Ingenuity pathway analysis
LOQ	Limit of quantification
MeHg	Methylmercury
mTOR	Mammalian target of rapamycin
RICTOR	Rapamycin-insensitive binding partner of mTOR
ROS	Reactive oxygen species
SeMet	Selenomethionine
